# Transient ischaemic attack due to incidental late electrical isolation of the left atrial appendage: a case report

**DOI:** 10.1093/ehjcr/ytaf622

**Published:** 2025-11-28

**Authors:** Sayana Kuraoka, Masatsugu Nozoe, Tomoki Uchikawa, Daisuke Nagatomo, Toru Kubota

**Affiliations:** Division of Cardiology, Cardiovascular and Aortic Center, Saiseikai Fukuoka General Hospital, 1-3-46, Tenjin, Chuo-ku, Fukuoka 810-0001, Japan; Division of Cardiology, Cardiovascular and Aortic Center, Saiseikai Fukuoka General Hospital, 1-3-46, Tenjin, Chuo-ku, Fukuoka 810-0001, Japan; Division of Cardiology, Cardiovascular and Aortic Center, Saiseikai Fukuoka General Hospital, 1-3-46, Tenjin, Chuo-ku, Fukuoka 810-0001, Japan; Division of Cardiology, Cardiovascular and Aortic Center, Saiseikai Fukuoka General Hospital, 1-3-46, Tenjin, Chuo-ku, Fukuoka 810-0001, Japan; Division of Cardiology, Cardiovascular and Aortic Center, Saiseikai Fukuoka General Hospital, 1-3-46, Tenjin, Chuo-ku, Fukuoka 810-0001, Japan

**Keywords:** Atrial fibrillation, Electrical isolation of left atrial appendage, Percutaneous left atrial appendage occlusion, Radiofrequency catheter ablation, Thrombo-embolic complication, Case report

## Abstract

**Background:**

Electrical isolation of the left atrial appendage (LAA) during catheter ablation may increase the risk of thrombo-embolism, even in patients with low stroke risk scores and sinus rhythm. Late incidental isolation of the LAA has been rarely reported as a cause of transient ischaemic attack (TIA).

**Case summary:**

A 73-year-old woman with a CHADS2 score of 0 underwent three catheter ablation sessions for long-standing persistent atrial fibrillation. Incomplete anterior mitral line ablation during the first and second sessions led to recurrent mitral flutter. At the third session, mitral flutter was successfully terminated by linear ablation at the left mitral isthmus, and electrical conduction to the LAA remained via the anterior LA wall. Anticoagulation was discontinued post-ablation. Five years later, she developed a TIA despite maintaining sinus rhythm. Four years after the TIA, she presented with atrial tachycardia. Voltage mapping during a fourth ablation revealed extensive anterolateral LA scarring and the absence of LAA electrograms, consistent with late incidental LAA isolation. Given her thrombo-embolic risk, percutaneous LAA closure with a WATCHMAN FLX device was successfully performed.

**Discussion:**

This case suggests that linear ablations forming a substrate around the LAA may lead to its progressive electrical isolation over time, increasing thrombo-embolic risk despite sustained sinus rhythm and low stroke risk scores.

Learning points

- Late electrical isolation of the left atrial appendage (LAA) can develop years after linear ablation, even without atrial fibrillation recurrence.
- LAA isolation may increase thrombo-embolic risk despite a low CHA₂DS₂-VASc score.
- It is generally safer to avoid combining the anterior mitral line and lateral mitral isthmus line, as this may predispose to progressive LAA isolation.

## Introduction

Ischaemic stroke and transient ischaemic attack (TIA) are serious risks in atrial fibrillation (AF) patients. Radiofrequency catheter ablation (RFCA) is effective for treating antiarrhythmic drug-refractory AF, and retrospective analyses suggest it reduces ischaemic stroke risk in AF patients.^1,2^ However, electrical disconnection of the left atrial appendage (LAA) may increase thrombo-embolic risk. We report a TIA case due to incidental late electrical isolation of the LAA.

## Summary figure

**Figure:**
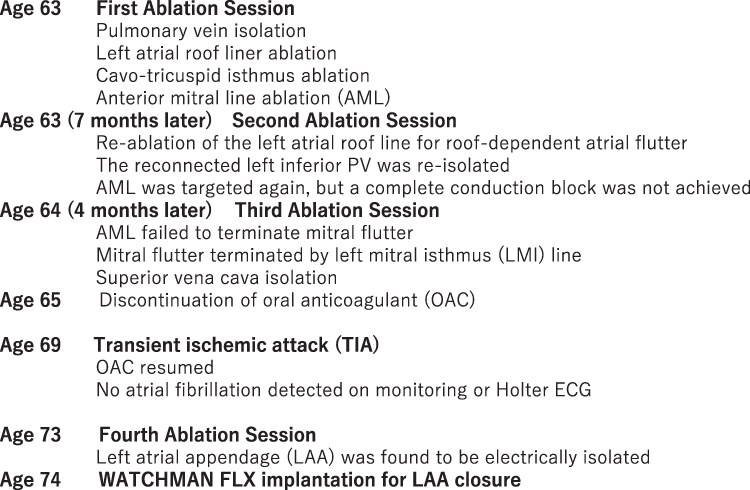


## Case presentation

**Figure 1:**
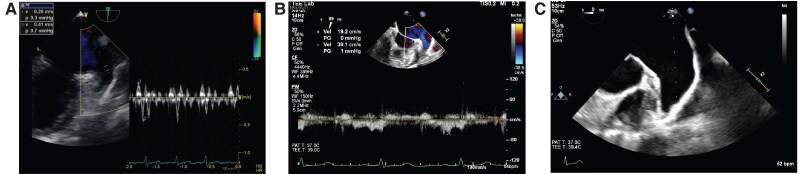
(*A*) Pre-operative transoesophageal echocardiography image at the first session. No thrombus inside the left atrial appendage was detected. Pulse Doppler showed that the average left atrial appendage flow velocity was 33.5 cm/s (emptying velocity 26.0 cm/s, filling velocity 41.0 cm/s). (*B*) Pulse Doppler image obtained by transoesophageal echocardiography at the fourth session. Average left atrial appendage flow velocity was decreased to compare with previous examination (average velocity 23.4 cm/s, emptying velocity 19.5 cm/s, and filling velocity 27.3 cm/s). (*C*) Transoesophageal echocardiography image of the left atrial appendage at the fourth session. No thrombus nor spontaneous echo contrast was detected.

A 63-year-old woman with persistent AF underwent her first catheter ablation. Transthoracic echocardiography (TTE) showed a LA diameter of 44 mm, LA volume of 73 mL (51 mL/m^2^), and preserved ejection fraction. Transoesophageal echocardiography (TEE) showed no LAA thrombus, with an average flow velocity of 33.5 cm/s (*Figure 1A*). Her CHADS2 and CHA2DS2-VASc scores were 0 and 1, respectively. Pulmonary vein (PV) isolation, cavotricuspid isthmus ablation, and LA roof linear ablation were performed with the CARTO system (Johnson & Johnson, New Brunswick, NJ), but AF/atrial tachycardia (AT) persisted. Given frequent recordings of complex fractionated potentials at the anterior wall of the LA during AT/AF, the anterior mitral line (AML) was created from the mitral annulus to the right superior PV. After several energy applications, cardioversion restored sinus rhythm, and she was discharged on sinus rhythm with bepridil 150 mg daily.

Seven months after the first session, due to palpitations and syncope, the second session was conducted. Linear ablation on the LA roof successfully terminated AT, and the reconnected left inferior PV was re-isolated. The AML was also targeted again, but a complete conduction block could not be achieved.

**Figure 2:**
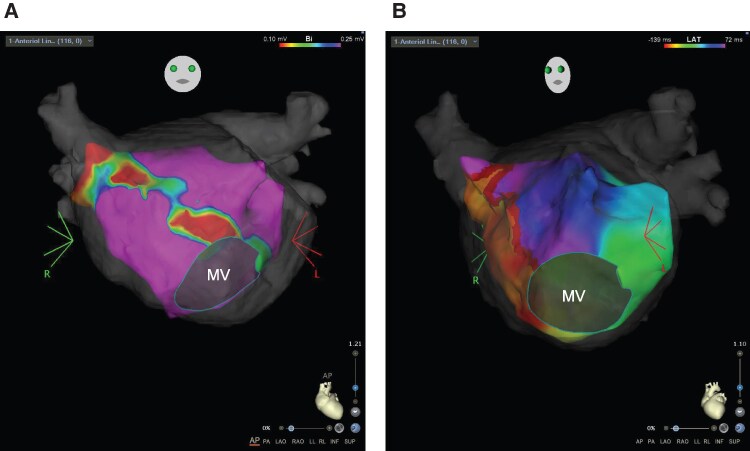
(*A*) Voltage mapping during atrial tachycardia using the CARTO system (Johnson & Johnson, New Brunswick, NJ) at the third session. Voltage mapping during atrial tachycardia revealed that the fragmented low-voltage electrograms were recorded in the anterior left atrium. (*B*) Activation mapping of the atrial tachycardia at the third session. Three-dimensional mapping using the CARTO system diagnosed the tachycardia as a counterclockwise mitral flutter. MV, mitral valve.

**Figure 3:**
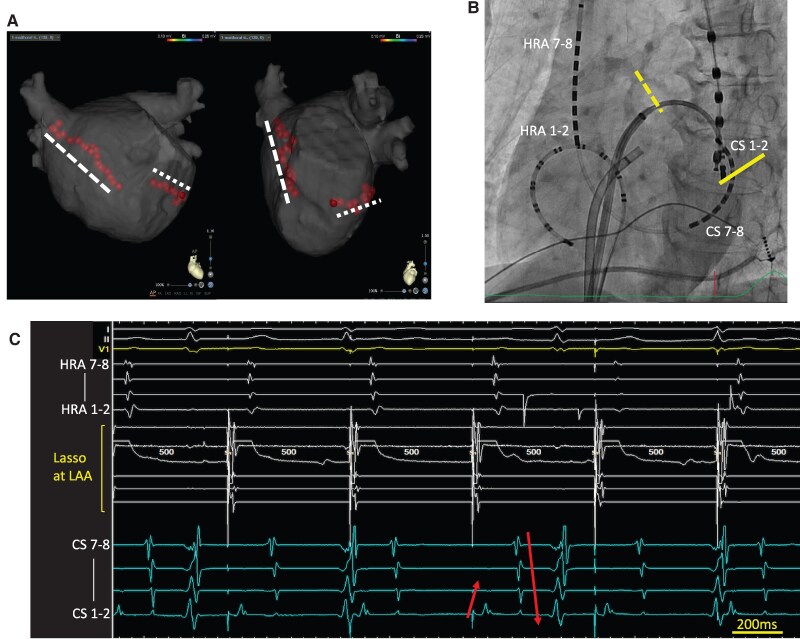
(*A*) Three-dimensional computed tomography images integrated into the CARTO system at the third session. Liner ablation at the anterior mitral line could not terminate tachycardia (dashed line). Therefore, we changed the strategy to make an electrical block line at the left mitral isthmus from the mitral annulus to the left inferior pulmonary vein (dotted line). Liner ablation at the left mitral isthmus successfully terminated the tachycardia. (*B*) Fluoroscopic view of the left anterior oblique 50°. Liner ablation at the anterior mitral line could not terminate tachycardia (dashed line). Therefore, we changed the strategy to make an electrical block line at the left mitral isthmus from the mitral annulus to the left inferior pulmonary vein (straight line). Liner ablation at the left mitral isthmus successfully terminated the tachycardia. (*C*) Electrocardiogram after successful ablation at the left mitral isthmus. Left atrial appendage pacing by a Lasso catheter (Johnson & Johnson, New Brunswick, NJ) revealed an electrical block line at the left mitral isthmus (arrow). The electrical connection towards the left atrial appendage was preserved through the left atrial anterior wall because the anterior mitral line was incomplete. The right atrium (7–8 proximal and 1–2 distal) and the coronary sinus (7–8 proximal and 1–2 distal) are shown from top to bottom at a paper speed of 100 mm/s.

She underwent the third session 4 months after the second session due to persistent symptoms. Holter electrocardiogram (ECG) revealed AT and paroxysmal AF followed by sinus arrest exceeding 5 s at tachycardia cessation. Three-dimensional mapping diagnosed the tachycardia as counterclockwise mitral flutter (*Figure 2A* and *B*). As ablation at the AML failed to terminate the mitral flutter, ablation was shifted to the left mitral isthmus (LMI) from the mitral annulus to the left inferior PV (*Figure 3A* and *B*). Mitral flutter was successfully terminated, and a bi-directional block at the LMI was confirmed via LAA pacing (*Figure 3C*). Observed firing from the superior vena cava (SVC) led to SVC isolation. No arrhythmias were inducible post-procedure, and the electrical connection to LAA remained connected through the incomplete AML. After the third session, she experienced symptom relief, maintaining sinus rhythm off antiarrhythmic drugs; oral anticoagulation (OAC) was discontinued 1 year later.

Five years after the third session, she developed a TIA with left hemiparalysis and numbness, improving within 90 min. Magnetic resonance imaging (MRI) initially showed no infarction, but follow-up revealed a subcortical infarction in the right temporal lobe. No AF recurrence was detected on monitor ECG or Holter monitoring. TTE showed normal systolic function, mild mitral regurgitation, LA diameter of 37 mm, and LA volume of 66 mL (47 mL/m^2^). Anticoagulation was resumed with dabigatran 300 mg daily, though she declined an implantable loop recorder. Sinus rhythm was maintained for several years.

**Figure 4:**
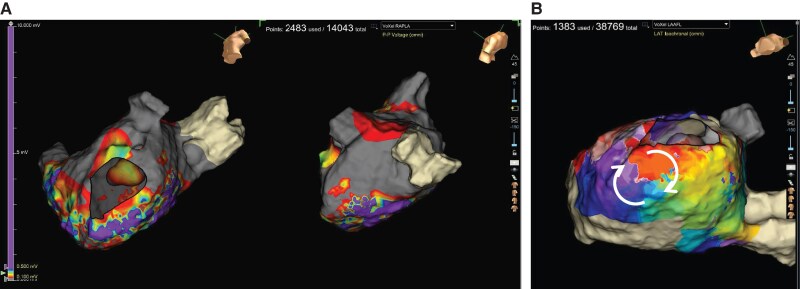
(*A*) Voltage mapping using Ensite X (Abbott, Chicago, IL) at the fourth session. Voltage mapping during right atrium pacing revealed a large scar at the anterolateral segment of the left atrium including the left atrial appendage. (*B*) Activation mapping of induced tachycardia. Atrial tachycardia (cycle length 226 ms) was diagnosed as localized re-entrant tachycardia at the inferior left atrium. The speculated atrial activation sequences are indicated by arrows.

Four years after TIA, she again experienced palpitations and presyncope with AT on ECG. The fourth session revealed extensive scarring in the anterolateral LA, including the LAA (*Figure 4A*), and no LAA electrograms were detected. The induced tachycardia was diagnosed as localized re-entrant tachycardia in the inferior LA, which was successfully terminated by energy application at the re-entrant site (*Figure 4B*). TEE after a fourth ablation showed reduced LAA flow velocity (average velocity 23.4 cm/s) without spontaneous echo contrast or thrombus (*Figure 1B* and *C*). Given her thrombo-embolic risk, percutaneous LAA closure with a 35 mm WATCHMAN FLX (Boston Scientific, Natick, MA, USA) device was successfully performed.

## Discussion

This case highlights a rare TIA associated with late incidental isolation of the LAA in a patient with low CHADS₂ and CHA₂DS₂-VASc scores. LAA isolation is a known complication during ablation procedures for atrial arrhythmias. Ghannam *et al*.^3^ reported that among 41 patients who experienced LAA isolation during a redo procedure, 37 (90%) had undergone additional linear ablation at the LMI (*n* = 34) and/or the AML (*n* = 13) to treat peri-mitral re-entry. In contrast to their findings, our patient did not have LAA isolation during the ablation procedures. The LAA remained electrically connected at the time, but gradual structural remodelling of the LA, likely promoted by age-related degeneration and the prior incomplete AML ablation, may have resulted in delayed isolation years later. This highlights the possibility that even when the LAA remains connected acutely, the combination of multiple linear ablations and subsequent structural remodelling may result in late isolation. Two liner ablations that sandwich the LAA should be avoided even if one of these were incomplete.

Impaired LAA contractility from an electrically disconnected LAA may increase the risk of thrombo-embolism. Recent studies have shown an increased incidence of ischaemic stroke following LAA isolation, with higher rates of thrombo-embolic events observed even in patients with incidental isolation.^3–6^ Additionally, wide isolation of the LAA, especially beyond the LAA base, is associated with a higher incidence of thrombus formation compared to distal LAA isolation.^4^ In the present case, electrical activity was absent in a large area of the lateral LA, including the LAA, and thrombo-embolism occurred despite a low CHA_2_DS_2_-VASc score.

Predicting ischaemic events in patients with incidental LAA isolation remains challenging. Ghannam *et al*.^3^ reported that six of seven patients (86%) with thrombo-embolism were in sinus rhythm at the time of the event after incidental LAA isolation. Among patients without LAA isolation, successful maintenance of sinus rhythm was associated with a significantly lower risk of ischaemic stroke or TIA, while the absence of late recurrence did not reduce ischaemic events in patients with LAA isolation.^5^ The LAA flow velocity, assessed by TTE, is a reliable marker of LAA contractility, with reduced velocities often seen in LAA-isolated patients compared to those without isolation.^4,5^ However, some studies report that patients with LAA thrombus formation or ischaemic events may not always demonstrate reduced flow velocities compared to patients without such events.^4,5^ Thus, LAA flow velocity alone may be insufficient to predict future ischaemic events.

Lifelong OAC therapy may be recommended for patients with LAA isolation, regardless of their CHADS_2_ score, although thrombo-embolic events can still occur under appropriate OAC therapy.^4,6^ Rillig *et al*.^4^ reported that 10 of 47 (21.3%) patients with LAA isolation developed LAA thrombus on TEE despite continuous OAC use in 90% of cases. Thus, LAA closure after LAA isolation may be a viable option to reduce thrombo-embolic risk without intensifying OAC and its associated risks. Heeger *et al*.^6^ reported reduced thrombo-embolic events in patients undergoing endocardial LAA closure post-isolation. Additionally, the WATCHMAN device has been demonstrated in multiple trials to be a reasonable alternative to OAC therapy for reducing thrombo-embolic risk.^4,7^ Panikker *et al*.^8^ simultaneously performed LAA electrical isolation and WATCHMAN closure in 20 patients undergoing RFCA for long-standing persistent AF, and the WATCHMAN device was successfully implanted in all patients without peri-procedural complications. Among them, 19 patients (95%) discontinued warfarin within 3 months post-ablation, and no TIA or stroke occurred. To eliminate the need for lifelong anticoagulation, a LAA closure device could potentially be used. More studies are required to assess whether we should occlude the LAA in all patients after its electric isolation.^9^

In principle, efforts should be made to avoid LAA isolation during AF ablation. However, if LAA isolation is unavoidable, or in patients at risk of isolation, LAA closure using the WATCHMAN device should be considered, regardless of their CHADS2 (or CHA2DS2-VASc) score.

## Data Availability

Data sharing is not applicable to this article as no datasets were generated or analysed during the current study.

## Lead author biography

Dr Sayana Kuraoka is a cardiology resident at Saiseikai Fukuoka General Hospital in Fukuoka, Japan. Her clinical and research interests include atrial fibrillation, catheter ablation, and stroke prevention. She is particularly focused on the long-term outcomes of left atrial appendage isolation and closure strategies. Dr Kuraoka is committed to advancing patient-centred electrophysiological care and plans to pursue further specialization in cardiac electrophysiology.

**Consent:** The authors confirm that written consent for submission and publication of this case report including images and associated text has been obtained the patient.

**Funding:** None declared.
